# Volume Thirteen

**Published:** 1873-08

**Authors:** 


### Editorial.

### Volume Thirteen.

With the present number we commence our thirteenth volume, trusting to the profession for help to make it more intrusting and instructive than any of its predecessors. The Editor has not been able for the last few months on account of illness, to give the JOURNAL much personal attention, but with comparatively restored health, now hopes for renewed and more satisfactory labor. To select from Medical literature the true and valuable,—the practically good, and reject the improbable, untrue and worthless requires much knowledge and experience and is a task which if poorly performed makes a JOURNAL worthless.

It has been our aim to furnish a reliable and practically instructive JOURNAL and our success though far short of what we hoped, has yet been exeedingly gratifying and satisfactory, and we now look back upon the twelve years of editorial labor and take pride in seeing how much we have been endorsed and sustained by the profession.

Those active thinking men who have furnished us so many original papers, some of them of great merit, will please accept our warmest thanks and an urgent invitation to continue the highly prized favor. We have but little to promise; the future is all unknown; successes and failures reveal themselves by time; it is well to hope, not wise to dread.

THE BUFFALO MEDICAL AND SURGICAL JOURNAL hopes to grow better, more instructive and more entertaining as it grow older, but what is old is not always true, and what is new is not always better than the old and tried; only by combining the wisdom of age with the progress of youth and vigor, will it fully deserve the favors it has thus far received. Hoping to merit approval we shall rely upon the profession for help to make the JOURNAL all that can be required of it.

